# Molecular xenomonitoring for post-validation surveillance of lymphatic filariasis in Togo: no evidence for active transmission

**DOI:** 10.1186/s13071-017-2611-9

**Published:** 2018-01-23

**Authors:** Monique A. Dorkenoo, Dziedzom K. de Souza, Yao Apetogbo, Komla Oboussoumi, Degninou Yehadji, Mawèke Tchalim, Santrao Etassoli, Benjamin Koudou, Guillaume K. Ketoh, Yao Sodahlon, Moses J. Bockarie, Daniel A. Boakye

**Affiliations:** 1 Faculté des Sciences de la santé, University of Lomé, BP 1515 Lomé, Togo; 2 Programme National d’Elimination de la Filariose Lymphatique, Ministère de la Santé et de la Protection Sociale, Angle avenue Sarakawa et avenue du 24 Janvier, BP 336 Lomé, Togo; 3 Department of Parasitology, Noguchi Memorial Institute for Medical Research, University of Ghana, Accra, Ghana; 4 Department of Animal Biology, Unité de Recherche en Ecotoxicologie, University of Lomé, BP 1515 Lomé, Togo; 5 Liverpool School of Tropical Medicine, Pembroke Place, Liverpool, L3 5QA UK; 6 Mectizan Donation Program, 325 Swanton Way, Decatur, Ga 30030 USA; 7 European & Developing Countries Clinical Trials Partnership (EDCTP), Medical Research Council, P.O. Box 19070, Cape Town, South Africa

**Keywords:** Lymphatic filariasis, Molecular xenomonitoring, Post-validation surveillance, Togo

## Abstract

**Background:**

Lymphatic filariasis (LF) is a mosquito-borne filarial disease targeted for elimination by the year 2020. The Republic of Togo undertook mass treatment of entire endemic communities from 2000 to 2009 to eliminate the transmission of the disease and is currently the first sub-Saharan African country to be validated by WHO for the elimination of LF as a public health problem. However, post-validation surveillance activities are required to ensure the gains achieved are sustained. This survey assessed the mosquito vectors of the disease and determined the presence of infection in these vectors, testing the hypothesis that transmission has already been interrupted in Togo.

**Method:**

Mosquitoes were collected from 37 villages located in three districts in one of four evaluation units in the country. In each district, 30 villages were selected based on probability proportionate to size; eight villages (including one of the 30 villages already selected) where microfilaremia-positive cases had been identified during post-treatment surveillance activities were intentionally sampled. Mosquitoes were collected using pyrethrum spray collections (PSC) in households randomly selected in all villages for five months. In the purposefully selected communities, mosquitoes were also collected using human landing collections (HLC) and exit traps (ET). Collected mosquitoes were identified morphologically, and the identification of Wuchereria bancrofti DNA in the mosquitoes was based on the pool screening method, using the LAMP assay.

**Results:**

A total of 15,539 mosquitoes were collected during the study. *Anopheles gambiae* (72.6%) was the predominant LF vector collected using PSC. Pool screen analysis of 9191 *An*. *gambiae* in 629 pools revealed no mosquitoes infected with *W. bancrofti* (0%; CI: 0–0.021).

**Conclusions:**

These results confirm the findings of epidemiological transmission assessment surveys conducted in 2012 and 2015, which demonstrated the absence of LF transmission in Togo. The challenges of implementing molecular xenomonitoring are further discussed.

## Background

Lymphatic filariasis (LF), the second leading infectious cause of disability worldwide, is endemic in 73 countries, an estimated 120 million people are infected with the parasites, and 40 million people suffer from complications. The World Health Organization (WHO) outlines specific steps for endemic countries to achieve and document interruption of transmission of LF [1]. Baseline mapping is conducted to identify LF-endemic areas of the country, followed by at least five rounds of annual mass drug administration (MDA) in endemic areas. Concurrent monitoring and evaluation (M&E) are conducted in these areas under MDA to ensure that targets are met and determine when MDA can be stopped. Transmission assessment surveys (TAS) are undertaken after MDA has been stopped, followed by a five-year post-validation period to verify the absence of resurgence.

Togo is a West African country with a population of 6.7 million, with 32% of the population living below the poverty line [2]. The country is divided into six regions and 40 health districts. District medical teams support more than 650 rural health centres nationwide. Eight of the 40 districts were endemic to LF. Those districts belong to three distinct endemic foci and were co-endemic for LF and onchocerciasis.

Togo was one of the first African countries to implement a national LF elimination programme [3]. The national LF programme started in 2000 with baseline mapping followed by mass drug administration in the LF endemic districts. The last MDA in Togo was conducted in 2009. LF surveillance in Togo has consisted of:

- a nationwide passive surveillance system, implemented from 2006 through 2015. The surveillance consisting of two components: (i) a network of 47 laboratories, in which laboratory technicians routinely search for *Wuchereria bancrofti* microfilaria on nocturnal blood smears collected for malaria diagnosis, and (ii) a network of 20 healthcare facilities not covered by the laboratory network, in which nurses regularly collect dried blood that is tested for Og4C3 antigen [4].
- a successful implementation of two post-MDA transmission assessment surveys (TAS) using the WHO’s guidelines in the eight endemic districts in 2012 and 2015, three and five years after stopping MDA, respectively, have been carried out.

The results of these surveillance activities demonstrated that there was no ongoing LF transmission in the country [3, 5].

Togo also conducted a national campaign to distribute insecticide-treated bed nets (ITNs) in 2004 followed by subsequent distribution of long-lasting insecticide-treated nets (LLINs) to achieve universal coverage [6–8]. These campaigns likely significantly changed the profile of the LF vector population in Togo, although entomologic studies are lacking.

Recently, Togo became the first country in sub-Saharan Africa to receive WHO validation of the elimination of LF as a public health problem [1, 9, 10]. Before this announcement, the country began molecular xenomonitoring (MX) for LF [11], to collect more evidence demonstrating the absence of LF transmission. This work, presented here, investigated the absence of LF transmission in mosquito populations as recommended by the WHO, with the hypothesis that transmission has already been interrupted in Togo [12].

## Methods

### Study design

The study had limited funding, and as such, it was important to consider various factors in developing the sampling. The development of the sampling strategy took place during a two-day meeting with the LF Programme officers and entomologists. Following extensive discussions, the least expensive option of using community volunteers as against trained entomologists was adopted for the study.

### Factors considered in designing the sampling strategy

The factors we considered in designing the sampling strategy include financial and human resources, workload and the local epidemiological context.

Financial resource factors affecting the sampling strategy are: (i) available budget; (ii) distances to be covered and associated transportation costs; (iii) daily allowances for entomologists, community volunteers and supervisors; and (iv) sourcing and purchasing of consumables and supplies.

Human resource factors considered are: (i) availability and skills of experienced entomologists versus community volunteers; (ii) need for supervision of volunteers during collections; (iii) training requirements (best practices for mosquito collection and storage) and logistics (training venues, meals and transportation allowances); (iv) need to develop human capacity for future public health needs; and (v) security and safety of trained entomologists.

Sampling decisions were also based on workload: (i) one-time versus repeated mosquito collections (i.e. probability of catching an infected mosquito in a one-time collection as compared to repeated collections); (ii) number of households that can be sampled per day; and (iii) total number of mosquitoes required.

Finally, the regional, national, and local epidemiological context affecting sampling are related to: (i) vector transmission dynamics (impact of seasonality on mosquito species and LF transmission); (ii) number of transmission assessment survey (TAS) evaluation units (EU) to investigate (weighing relative benefits of sparse coverage of all EUs against comprehensive coverage of one EU); and (iii) relative emphasis on high-risk versus low-risk areas in the sample.

### Study area

**Fig. 1:**
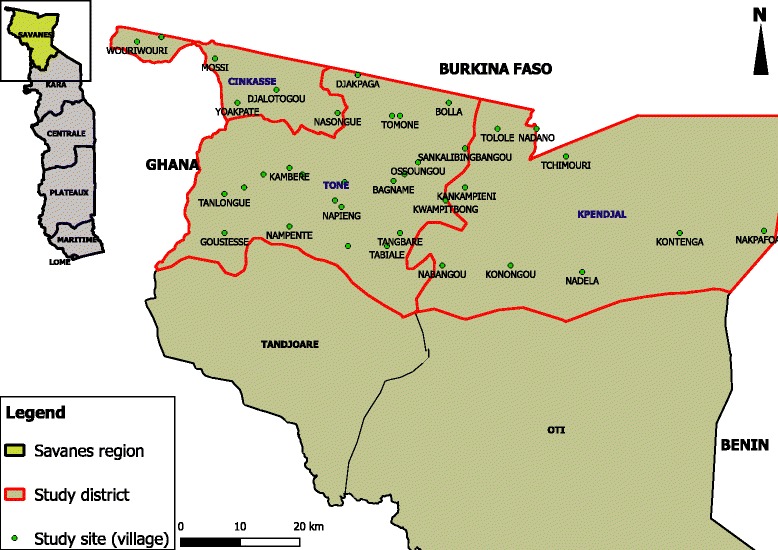
Study region and communities. Base map source: Institut national de la Statistique, des études économiques et démographiques, Togo (INSEED). Software: QGIS 2.18.14 (Las Palmas) (Quantum GIS Geographic Information System, Quantum GIS Development Team, Open Source Geospatial Foundation Project)

The study was conducted in the Savanes Region in the northern part of Togo (Fig. 1). Three of the five districts in the region, Kpendjal, Cinkassé and Tone, were previously endemic for LF. The survey was conducted in the three districts grouped into one evaluation unit (EU) because of their proximity to Ghana in the West, Burkina Faso to the North and Benin to the East, where transmission of LF is reported to be ongoing.

### Selection of sampling sites

A two-stage sampling method was used to select the sampling sites. In the first stage, villages were chosen, and in the second, households (HHs) were selected within each village. [13]. All communities with population greater than 5000 were excluded from the sampling because the potential for transmission in urban areas is low [14, 15]. Thirty villages were selected in the EU with probability proportional to size.

In addition, eight additional villages previously known to have reported a microfilaremia positive case, either through monitoring and evaluation, TAS or passive surveillance, were also assessed. The last microfilaremia positive case was identified in 2015. One of the villages purposefully selected was also selected by probability proportionate to size; therefore, 37 villages were surveyed in total. The geolocation of each surveyed village was recorded.

Households were sampled to cover the entire village as much as possible, by the end of the sampling period. Each village was divided into four approximately equal sections. Households in each section were numbered consecutively and selected randomly (using a dice). The household head was approached, and consent sought. If a household refused to participate, a different household was selected. New households were selected for each sampling day, and households from which mosquitoes were previously collected were excluded from the selection unless the number of households in the section was exhausted.

### Mosquito collection

This entomological study was undertaken over five months during the peak dry season (October 2016–February 2017), with the aim of collecting as many mosquitoes as possible. In each study community, mosquito collection was done twice every month. The estimated sample size was 2000 vector mosquitoes per IU, required to estimate an infection rate of 1% with a power of 0.80 [16].

In each village, community volunteers were identified and trained for mosquito collection and storage. Mosquito collections were primarily done using the pyrethrum spray catch (PSC) method. On each mosquito collection day, households were randomly selected from each section and mosquitoes collected, using the PSC method. The day before the collection, consent was obtained from occupants of the households, they were asked to keep bedroom doors and windows closed the following morning. Mosquitoes were collected early in the morning between 05:00 h and 08:00 h by two trained collectors. The occupants were asked to remove or cover all food items in the room. Potential mosquito hiding places (under the bed, tables) were searched and disturbed to displace any resting mosquito White sheets were laid on the floor and other surfaces in the rooms. The room was then sprayed with pyrethrum insecticide and left for about 15 min, after which the white sheets were inspected for any dead or knocked down mosquitoes.

In the eight purposefully selected communities, mosquito collection was also done using human landing collection (HLC) method and exit trap collection (ETC). Both HLC and ETC were undertaken in randomly selected households, different from the households where PSC was undertaken.

All collected mosquitoes were placed in a Petri dish labelled with the village code. Each Petri dish contained silica gel in a ball of cotton wool, to keep the mosquitoes dry. The Petri dishes from all villages were sent to the district on a specified date. Once a month, a central team visited all districts to collect the mosquitoes and deliver them to the entomology laboratory of the department of “Unité de Recherche en Ecotoxicologie (URET)” of Sciences faculty of the University of Lomé (Togo).

### Sample processing

**Fig. 2:**
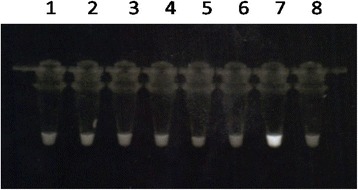
The reaction tubes after LAMP reaction. The positive control (Tube 7) turned white when visualized under a UV source. The negative samples (tubes 1–6) and negative control (tube 8) are clear

Mosquito genera and species identification were conducted at the entomology laboratory of the University of Lomé using morphological identification keys [17, 18]. All mosquitoes collected by the PSC and ETC were grouped in pools of 25 or less, according to the village and method of collection. The pooled mosquitoes were then sent to the NTD reference laboratory of the Parasitology Department, Noguchi Memorial Institute for Medical Research (University of Ghana) for molecular identification of *W. bancrofti* infection in the vector species. DNA was extracted from the mosquito pools using the DNeasy Tissue Kit (Qiagen, Valencia, California, USA), and molecular identification of *W. bancrofti* was done using the LAMP method [19–21]. All reactions included a positive (*W. bancrofti* DNA) and negative (water) control (Fig. 2) The positive control is *W. bancrofti* DNA extracted from microfilariae positive mosquitoes, in previous studies from Sierra Leone [20]. All positives were confirmed using the conventional PCR method for the determination of *W. bancrofti* infection [22].

### Data analysis

The number of mosquitoes collected in each month was evaluated regarding the rainfall data for each collection month. The Poolscreen 2.0 software was used to analyse the pool screening results [23]. The survey costs were grouped into categories and presented in a table. Survey costs were divided into the following categories: personal allowance (research team and vector collectors), supplies, transportation, communication, and others. The unit cost per sample was estimated as the sum of the unit cost for collecting each sample and the unit cost for laboratory analysis.

All levels of statistical significance were determined at the 95% confidence limit. Graphs were drawn using GraphPad Prism 7 (GraphPad Software, Inc., La Jolla, California, USA) and Microsoft Excel (Microsoft Corporation, Redmond, Washington, USA). The geolocation data were imported into QGIS 2.18 (QGIS Geographic Information System, QGIS Development Team, Open Source Geospatial Foundation Project) for mapping.

## Results

A total of 15,568 mosquitoes were collected over the entire 5-month period: 10,859 by PSC, 3798 by ETC and 911 by HLC. Table 1 presents the number of mosquitoes per collection method and IU. More mosquitoes were collected using the PSC.

**Table 1:** Number and species of mosquitoes by collection method and district

Method	District	No. of communities	*An. gambiae*	Mean *An. gambiae*	*Culex* spp*.*	*Ae. aegypti*	*Mansonia* spp*.*	Other species
PSC	Tone	21	3956	188	1890	8	1	6
	Cinkasse	6	1885	314	620	7	2	0
	Kpendjal	10	2052	205	432	0	0	0
	Total	37	7893	213	2942	15	3	6
ETC	Tone	3	1008	48	970	25	7	0
	Cinkasse	3	683	114	36	9	18	0
	Kpendjal	2	642	64	368	13	19	0
	Total	37	2333	63	1374	47	44	0
HLC	Tone	3	329	110	160	6	1	0
	Cinkasse	3	214	71	16	7	7	0
	Kpendjal	2	113	57	57	1	1	0
	Total	8	656	82	233	14	9	0

*Anopheles gambiae* (*s*.*l*.) (*n* = 7893), the main vectors of LF in West Africa, was the dominant mosquito species (72.6%) collected using the PSC, followed by *Culex quinquefasciatus* (*n* = 2942), *Aedes* (*n* = 15), *Mansonia* (*n* = 3) and six other mosquito species. The mean number of *An. gambiae* collected per village using the PSC was 213. For the ETC, 2333 *An. gambiae*, 1374 *C. quinquefasciatus*, 47 *Aedes* and 44 *Mansonia* were collected. The mean number of *An. gambiae* collected per village using ETC was 63. For HLC, a total of 656, 233, 12 and 10 mosquitoes were reported for the *An. gambiae*, *C. quinquefasciatus*, *Aedes*, and *Mansonia* species, respectively. The mean number of *An. gambiae* collected per village using HLC was 82. Based on HLC, the overall biting rate was at the highest in October 2016, with 23 bites/person/night (bpn) for indoor and 22 bpn for outdoor collections, and lowest in February 2017 with 1 bpn for both indoor and outdoor collections.

*Anopheles* spp. were processed for *W. bancrofti* infection. Out of 9191 samples processed in 629 pools, none (0%) were found positive (95% CI: 0–0.021). Table 2 presents the information on the number of pools analysed per IU. Of the mosquitoes analysed, 7623 were either fed or gravid, and 1568 were unfed. Of the *An. gambiae* collected, 6992 were analysed using PSC, and the remainder using ETC.

**Table 2:** Number of mosquitoes and pools processed per district

Implementation unit	No. of pools	Average pool size	No. of *An. gambiae* processed	No. of *An. gambiae* collected	% of *An. gambiae* processed	Positive	95% CI
Cinkasse	166	15.9	2646	2782	95.1	0	0–0.073
Kpendjal	172	14.5	2495	2807	88.9	0	0–0.077
Tone	291	13.9	4050	5283	76.7	0	0–0.047
Total	629	14.6	9191	10,872	84.5	0	0–0.021

*Abbreviation*: CI, confidence interval

The costs of this survey are summarized in Table 3, with the aim of guiding other control programmes planning to undertake wide-scale entomological evaluations for LF elimination. An estimated $35,910.40 was spent on this survey. The main costs incurred include the training of the community mosquito collectors and supervisors, field mosquito collection (person-time), and sample processing charges. Other costs included ethical application fees, photocopies and vehicle maintenance charges. Based on the cost estimates for the study, the approximate costs for sample collection per IU, cost for sample collection per week, cost per pool collected and processed and cost per mosquito collected and processed were estimated. The cost per mosquito collected and processed was estimated at 3.1 US$.

**Table 3:** Cost of survey

Item		Cost (CFA Francs)	Approximate cost (US$)	%
Consultancy	Per diem	500,000.0	874.10	2.43
	Transport	120,000.0	209.800	0.58
Training	Central level per diem	1,127,000.0	1970.3	5.49
	Fuel	298,905.0	522.60	1.46
	Allowance for supervisors and mosquito collectors	568,000.0	993.00	2.77
	Transport for participants	364,000.0	636.40	1.77
	Training venues and meals	356,750.0	623.70	1.74
Field collection	Central level per diem	1,818,000.0	3178.30	8.85
	Allowances for collectors and supervisors	3,465,000.0	6057.70	16.87
	Transport for supervisors	376,275.0	657.80	1.83
	Field supplies	2,835,443.0	4957.10	13.80
	Other consumables	255,500.0	446.70	1.24
	Fuel	617,000.0	1078.70	3.00
	Others	98,350.0	171.90	0.48
Sample processing	Mosquito identification	676,000.0	1181.80	3.29
	Shipment of samples	120,000.0	209.80	0.58
	Laboratory processing charges	5,968,120.0	10,433.80	29.05
Others expenses	Ethics application	200,000.0	349.70	0.97
	Vehicle maintenance	300,400.0	525.20	1.46
	Data management	200,000.0	349.70	0.97
	Communication	214,395.0	374.80	1.04
	Photocopies	61,630.0	107.70	0.30
Total		20,540,768.0	35,910.40	100.00
Summary Estimates				
~ Sample collection cost per district		8492.20 US$		
~ Sample collection cost per week		2547.70 US$		
~ Cost per pool collected and processed		45.40 US$		
~ Cost per mosquito collected and processed		3.10 US$		

## Discussion

In Togo, MDA conducted during the past ten years has dramatically reduced lymphatic filariasis (LF) incidence in all implementation units (IU) as demonstrated by the low prevalence of circulating filariasis antigenemia obtained during the two TAS conducted during the five years after MDA was stopped [3]. Despite the very low infection rate in the human population, and the prior submission to WHO of a dossier of evidence supporting the elimination of LF as a public health problem in Togo, this study of *W. bancrofti* in the vector was conducted to confirm the results of the epidemiological surveys. As WHO states, the establishment of post-validation surveillance is important to prevent recrudescence of infection and renewed transmission [9]. Efficient and sensitive methods are needed to detect this recrudescence risk, and molecular xenomonitoring is gaining recognition as one of the tools to be employed as a surveillance tool in the endgame [15, 21, 24–27].

The molecular analysis of the *An. gambiae* in this study revealed no infection with *W. bancrofti*, which is consistent with the very low antigen prevalence observed during the TAS conducted from 2010 to 2015 [3, 5, 28]. In this study, culicine mosquitoes were not analysed, based on the assumption that they are not known vectors of LF in West Africa [29]. In fact, studies conducted in Togo in the 1960s revealed *An. gambiae*, *An. funestus*, *An. pharoensis*, *Mansonia africana* and *M. uniformis* to be naturally infected with *W. bancrofti*. The authors suggested that only the first three species seemed to be vectors, with *An. gambiae* being a proven local vector of *W. bancrofti* in Togo [30]. Nonetheless, more recent surveys in Kano (Nigeria) revealed L3 *W. bancrofti* infection in *Culex quinquefasciatus*, with others also reporting infection and infectivity rates in *Culex* mosquitoes [29, 31, 32]. As such, in the light of changing vector abundance and transmission dynamics, future surveys should also focus on mosquito species previously considered as non-vectors, since parasite DNA can be detected in both vector and non-vector mosquitoes [21, 33].

WHO recommendations for the use of PCR on pools of mosquitoes for end-point assessment and post-MDA surveillance suggest that molecular xenomonitoring and mosquito sampling assessment should focus on individual villages (or a cluster of villages when villages are small), rather than on implementation units [16]. Here, the mosquitoes were processed according to the village and IU of the collection. However, whether processing approximately 200 mosquitoes per village (especially where microfilariae positive individuals were identified during surveillance) is enough to identify residual infection remains a matter of debate. Further statistics and modelling analyses might be required to resolve this challenge. While the sample sizes per IU were met, the possibilities of missing communities with the low residual transmission remain, since not all villages in the IUs were assessed. Implementing the WHO recommendation will require (i) more frequent mosquito collections per month in each community to have a large enough sample size, and (ii) mosquito collection in many more villages. These will ultimately increase the cost of xenomonitoring surveys. Finally, while the mosquitoes processed by pool screening were not presented according to catch type and species, due to the absence of infection, the number of mosquitoes collected per village using PSC was higher. A recent study showed that collecting mosquitoes using PSC may be a better tool for xenomonitoring compared to ETC [34]. As such further studies may focus on using PSC alone or other epidemiologically relevant tools such as gravid traps.

Given the very low parasite detection during TAS, we wanted to collect and analyse as many mosquitoes as possible, to enable the detection of very low *W. bancrofti* infection in the mosquitoes. While the survey was initially planned for all four EUs in the country, with a large number of sites per EU (for good coverage, considering that this was the first post-validation survey for the country), we ultimately could not achieve both with the available budget, so we elected to conduct the survey in many households but in only the highest risk EU. The cost for the survey covered by the programme was approximately 35,910.00 US$. The major cost components were the personnel (34%), laboratory processing (32.3%), field supplies (15%) and transportation costs (8.6%). It is, however, worth mentioning that additional reagents (~3000.00 US$) were donated, by the Noguchi Centre, to enable processing of as many samples as possible. Based on these, the mean approximated cost spent per village is 1051 US$ (including personnel, and sample processing costs). In designing the sampling strategy, various factors and scenarios were considered to arrive at the most cost-effective design. The preferred sampling strategy will differ from country to country, and therefore so will the cost differ [35].

## Conclusions

The post-validation molecular xenomonitoring survey in Togo has revealed the absence of infection in the *An. gambiae* vectors of LF, confirming the results of TAS and surveillance activities that led to the validation of elimination of LF as a public health problem in Togo. The application of molecular xenomonitoring was not without challenge. However, we have demonstrated that large-scale xenomonitoring is feasible. In addition to the challenges of protocol development, sample sizes for evaluation and availability of technical expertise will need to be addressed in order to enable Togo and other country programmes to integrate molecular xenomonitoring into their pre- and post-validation surveillance activities. A further challenge for Togo is to extend the same survey to the five remaining implementation units in the country. Given the proximity of areas of ongoing LF transmission in neighbouring countries, Togo will need to continue epidemiological and entomological monitoring and promote key prevention strategies, such as ITN use, to preserve its achievement and keep Togo a country where future generations can live free of LF.

## Abbreviations

ETC: exit trap collection
EU: evaluation unit
HH: household
HLC: human landing collection
ITN: insecticide treated net
IU: implementation unit
LAMP: loop mediated isothermal amplification
LLIN: long lasting insecticidal net
M&E: monitoring and evaluation
MDA: mass drug administration
MX: molecular xenomonitoring
NMIMR: Noguchi Memorial Institute of Medical Research
NTD: neglected tropical diseases
PSC: pyrethrum spray catch
TAS: transmission assessment survey
URET: Unité de Recherche en Ecotoxicologie
UV: ultraviolet
WHO: World Health Organization
